# Evidence from Cochrane systematic reviews for effects of antithrombotic drugs for lower-limb revascularization. A narrative review

**DOI:** 10.1590/1516-3180.2020.0640.110321

**Published:** 2021-08-13

**Authors:** Silfayner Victor Mathias Dias, Ronald Luiz Gomes Flumignan, Wagner Iared

**Affiliations:** I MD. Researcher, Postgraduate Program on Evidence-Based Health, Universidade Federal de São Paulo (UNIFESP), São Paulo (SP), Brazil.; II MD, PhD. Adjunct Professor, Division of Vascular and Endovascular Surgery, Universidade Federal de São Paulo (UNIFESP), São Paulo (SP), Brazil.; III MD, PhD. Advisory Professor, Postgraduate Program on Evidence-Based Health, Universidade Federal de São Paulo (UNIFESP), São Paulo (SP), Brazil.

**Keywords:** Peripheral arterial disease, Anticoagulants, Platelet aggregation inhibitors, Systematic review [publication type], Vascular grafting, Antiplatelet, Thromboses, Endovascular, Bypass

## Abstract

**BACKGROUND:**

Peripheral arterial disease (PAD) is characterized by progressive narrowing of the arterial lumen, resulting from atherosclerotic plaques. Treatment for PAD aims to control atherosclerosis and improve blood flow. Use of antiplatelet agents and anticoagulants has played important roles in helping to prevent occlusions and stenosis.

**OBJECTIVE:**

To evaluate the evidence from Cochrane systematic reviews regarding the accuracy, effectiveness and safety of use of anticoagulants and antiplatelets in lower-limb revascularization, in patients with peripheral arterial disease.

**METHODS:**

Systematic reviews found through searches in the Cochrane Library were included. Two authors evaluated whether the reviews found were in line with the inclusion criteria for this investigation. A qualitative synthesis of their findings was presented.

**RESULTS:**

Three systematic Cochrane reviews were included. Patients who underwent prosthetic bypass surgery probably presented greater benefit from use of antiplatelets, and patients who underwent vein revascularization probably presented greater benefit from use of anticoagulants. Patients who received endovascular treatment benefited from both antiplatelet and anticoagulant treatment. However, the reliability of the results found was impaired because at the time when these reviews were published, there was no mandatory assessment using the GRADE criteria.

**CONCLUSION:**

Despite the evidence found, it is necessary for these reviews to be updated in order to evaluate the degree of certainty of the results found.

## INTRODUCTION

Peripheral arterial disease (PAD) is characterized by progressive narrowing of the arterial lumen resulting from atherosclerotic plaques on the artery walls. The disease has a prevalence of 18% in 50-year-old patients, and this reaches 29% in patients over 70 years of age.^1^ Its incidence has been increasing over recent decades due to population growth, aging of the population and increased incidence of diabetes mellitus and smoking.^2^

These patients are at higher risk of mortality due to acute myocardial infarction (AMI) and stroke. Thus, treatment of PAD offers additional prevention for cardiovascular events.^3^

PAD commonly leads to intermittent claudication of the lower limbs, characterized by muscle pain during muscle activity, caused by restricted blood flow to the muscles recruited. This condition improves after a brief rest. As blood flow restriction increases, pain at rest and gangrene arise. In addition, these patients have significantly reduced quality of life due to restricted mobility.^4^

The aim in treating PAD is to control the risk factors for atherosclerosis and bring symptomatic relief through improving blood flow. In more advanced cases of the disease, revascularization of the lower limbs is required through surgery or percutaneous transluminal angioplasty.

Anticoagulant and antiplatelet agents play an important role in helping to prevent occlusions and stenosis, both for patients who have started treatment and for those undergoing revascularization, thus sustaining the clinical improvement.^5^^,^^6^ Platelets participate in the process of hemostasis and pathogenesis of atherosclerotic disease. The presence of endothelial injuries exposes the subendothelial extracellular matrix to contact with platelets.^7^ This mechanism promotes platelet recruitment, adhesion, activation and aggregation, to form a prothrombotic surface that promotes formation of clots and fibrous plaques and the ensuing thromboembolic complications. The latter can lead to acute myocardial infarction, stroke and peripheral vascular occlusions.^7^

## OBJECTIVES

The objective of this study was to summarize the evidence from Cochrane systematic reviews on the safety and effectiveness of use of antiplatelet agents and anticoagulants in lower limb revascularization in PAD.

## METHODS

### Study design and location

This was a narrative review of Cochrane systematic reviews developed within the Evidence-Based Health postgraduate program at Escola Paulista de Medicina (EPM), Universidade Federal de São Paulo (UNIFESP).

### Inclusion criteria

### Types of study

Only Cochrane systematic reviews were included, and only the latest version of the review was considered. Reviews that had been excluded from the Cochrane Library and systematic review protocols (reviews in progress) were not included.

#### Types of participants

We included reviews in which the participants presented the diagnosis of chronic PAD of the lower limbs and who had undergone revascularization of the lower limbs by means of venous or prosthetic bypasses or through angioplasty.

#### Types of interventions

We included studies in which patients underwent any treatment with antiplatelet agents or anticoagulants, and relevant outcomes were assessed in relation to the evolution of the disease.

#### Types of outcomes

Outcomes relating to the evolution of the disease and to morbidity, mortality and safety of treatments were considered.

### Search for studies

We performed a systematic search in the Cochrane Database of Systematic Reviews (via Wiley) on July 11, 2020. The search strategy is detailed in Table 1.

**Table 1:** Search strategy used in the Cochrane Library

Lines	Search terms	Numbers of records
#1	MeSH descriptor: [Peripheral Arterial Disease] explode all trees	1,039
#2	MeSH descriptor: [Arterial Occlusive Diseases] explode all trees	12,019
#3	MeSH descriptor: [Peripheral Vascular Diseases] explode all trees	3,211
#4	#1 OR #2 OR #3	13,967
#5	(Peripheral Arterial Disease*) OR (Arterial Occlusive Disease*) OR (Arterial Obstructive Disease*) OR (Vascular Disease*) OR (Peripheral Vascular Disease*) OR (Peripheral Angiopath*)	29,451
#6	MeSH descriptor: [Anticoagulants] explode all trees	4,596
#7	(Anticoagulant Agent*) OR (Anticoagulant Drug*) OR (Anticoagulant) OR (Indirect Thrombin Inhibitor*)	6,517
#8	MeSH descriptor: [Platelet Aggregation Inhibitors] explode all trees	3,870
#9	(Blood Platelet Antiaggregant*) OR (Platelet Antiaggregant*) OR (Platelet Aggregation Inhibitor*) OR (Blood Platelet Aggregation Inhibitor*) OR (Platelet Inhibitor*) OR (Antiplatelet Agent*) OR (Antiplatelet Drug*) OR (Platelet Antagonist*) OR (Blood Platelet Antagonist*)	11,027
#10	#4 OR #5	38,968
#11	#6 OR #7	9,747
#12	#8 OR #9	11,027
#13	#10 AND (#11 OR #12)	3,231
#14	in Cochrane Reviews	370

### Selection of studies

Two authors (SVMD and RLGF) evaluated and selected the titles and abstracts of systematic reviews regarding their agreement with the eligibility criteria of this study. Any occurrences of disagreements were resolved by a third researcher (WI).

### Presentation of results

The findings of the systematic reviews were summarized and presented narratively.

## RESULTS

The initial search resulted in retrieval of 370 systematic Cochrane reviews, of which three met the inclusion criteria for this review.^8^^–^^10^ We present, in summary, the main methodological characteristics and the most relevant results of the reviews included. In **Table 2**, we present a summary of the main findings. Reviews involving medications that are no longer available worldwide for clinical use were excluded from this study.

**Table 2:** Characteristics and main results of the systematic reviews on the clinical treatment of peripherical arterial disease of the lower limbs that were included

Review	Interventions	Main results
**Antiplatelet agents for preventing thrombosis after peripheral arterial bypass surgery** ^6^	ASA 400-990 mg/d or ASA 400-900 mg/d plus DIP 150-450 mg/d versus placebo or nothing	**Benefits with ASA or ASA plus DIP** Primary patency in venous bypass at 12 months Primary patency in prosthetic bypass at 1, 3, 6, 9 and 12 months
		**No difference among intervention groups** Gastrointestinal side effects; major bleeding; minor bleeding; wound or graft infection; limb amputation; cardiovascular events; mortality; primary patency in venous bypass at 1, 3, 6 and 24 months
		**Higher risk with ASA or ASA plus DIP** General side effects
	ASA or ASA 1050 mg/d plus DIP 150 mg/d versus pentoxifylline 1200 mg/d; venous or prosthetic bypasses	**Benefit with pentoxifylline** Less gastric intolerance
		**No difference among intervention groups** Primary patency at 1, 3, 6 and 12 months; gastric bleeding; dizziness; limb amputation; mortality
	ASA 900 mg/d plus DIP 250 mg/d versus indobufen 400 mg/d; venous or prosthetic bypasses	**No difference among intervention groups** Primary patency at 1, 3, 6, 9 and 12 months
	ASA 1000 mg or ASA 1000 mg plus DIP 225 mg/d versus VKA; venous or prosthetic bypasses	**No difference among intervention groups** Graft primary patency at 3, 6, 12 and 24 months; limb amputation; cardiovascular events; mortality
	ASA 900 mg/d plus DIP 300 mg/d versus LMWH 2500 IU/d; venous or prosthetic bypasses	**Benefits with ASA plus DIP** mortality
		**No difference among intervention groups** Graft primary patency at 6 and 12 months
	Ticlopidine 500 mg/d versus placebo; venous bypass	**Benefits with ticlopidine** Graft primary patency at 6, 12 and 24 months
		**No difference among intervention groups** Graft primary patency at 1 month
	ASA 1500 mg/d versus prostaglandins (PGE1) 0.2 ng/kg/min; vein bypass	**No difference among intervention groups** Early occlusion
	Clopidogrel 75 mg/d plus ASA 75-100 mg/d versus ASA 75-100 mg/d	**Benefits with clopidogrel plus ASA** Primary patency in prosthetic bypasses at 24 months; amputation in prosthetic bypasses
		**No difference among intervention groups** Amputation of venous bypasses; mortality for venous or prosthetic bypasses; primary patency in venous bypasses at 24 months; minor, mild or major bleeding for prosthetic bypasses; major bleeding for venous bypasses
		**Higher risk with clopidogrel plus ASA** Minor or mild bleeding for venous bypasses
**Antithrombotic agents for preventing thrombosis after infrainguinal arterial bypass surgery** ^7^	VKA versus placebo	**Benefits with VKA** Occlusion in venous bypasses at 6 months; occlusion in prosthetic bypasses at 5 years; limb loss in venous bypasses at 5 years; limb salvage for venous bypasses at 6 months; limb loss for venous or prosthetic bypasses at 3, 6 and 24 months and 5 years; mortality for venous or prosthetic bypasses at 12 months
		**No difference among intervention groups** Occlusion in venous bypasses at 3, 12 and 24 months and 5 years; occlusion in prosthetic bypasses at 3, 6, 12 and 24 months; limb loss in venous or prosthetic bypasses at 3, 6, 12, 24 months and 5 years for prosthetic; mortality in venous or prosthetic bypasses at 3, 6, 12 and 24 months and 5 years
	VKA versus ASA 80 mg/d or ASA 1000 mg/d plus DIP 225 mg/d	**Benefits with VKA** Occlusion in venous bypasses at 3, 6, 12 and 24 months
		**Benefits with ASA plus DIP** Occlusion in prosthetic bypasses at 6, 12 and 24 months
		**No difference among intervention groups** Occlusion in prosthetic bypasses at 3 months
	LMWH (enoxaparin) 40 mg versus UFH 5000 IU (intraoperative)	**Benefits with LMWH** Occlusion in venous or prosthetic bypasses at 10 and 30 days
		**No difference among intervention groups** Occlusion in venous or prosthetic bypasses at 24 hours
	LMWH (dalteparin) 2500 IU versus ASA 900 mg/d plus DIP 300 mg/d	**Benefits with LMWH** Occlusion in venous or prosthetic bypasses at 6 and 12 months
	LMWH (dalteparin) 5000 IU versus placebo	**No difference among intervention groups** Occlusion in venous or prosthetic bypasses at 1, 3 and 12 months
	UFH 5000 IU versus antithrombin 1500 IU	**Benefits with UFH** Intraoperative occlusion of venous or prosthetic bypasses.
		**No difference among intervention groups** Occlusion in venous or prosthetic bypasses at 1 month
	LMWH (enoxaparin) 40 mg/d versus dextran 2500 ml plus heparin 5000 IU	**No difference among intervention groups** Early occlusion in venous or prosthetic bypasses
**Antiplatelet and anticoagulant drugs for prevention of restenosis/reocclusion after peripheral endovascular treatment** ^8^	ASA 50-330 mg/d plus DIP 75-400 mg/d versus placebo	**Benefits with ASA (330 mg)/DIP** Occlusion/restenosis at 6 months
		**No difference found among interventions** Occlusion/ restenosis at 1, 3 and 6 months (other doses) and 12 months; amputation, mortality and bleeding at the puncture site at 1 month
		**Higher risk with ASA (1000 mg/d) plus DIP** Gastrointestinal side effects at 12 and 24 months
	ASA 150-990 mg/d plus DIP 225-400 mg/d versus VKA	**No difference found** Occlusion/restenosis at 1, 3, 6, 12, 24 and 36 months
	Clopidogrel 75 mg/d plus ASA 100 mg/d versus LMWH (dalteparin) 5000 IU followed by warfarin	**No difference found** Occlusion/restenosis at 24 hours and 1, 6, 12 and 18 months
		**Higher risk with LMWH followed by warfarin** Major bleeding
	Ticlopidine 1000 mg/d versus VKA	**No difference found** Occlusion/restenosis at 12 months
		**Higher risk with ticlopidine** Gastrointestinal side effects
	Cilostazol 200 mg/d plus ASA 100 mg/d versus ticlopidine 200 mg/d plus ASA 100 mg/d	**Benefits with cilostazol plus ASA** Occlusion/restenosis at 36 months.
		**No difference found** Occlusion/restenosis at 12 and 24 months; amputation, mortality and side effects at 36 months
	LMWH (therapeutic nadroparin) plus ASA 200 mg/d versus UFH (heparinization followed by ASA)	**Benefits with LMWH versus UFH** Occlusion/restenosis at 3 weeks and 3 and 6 months (femoropopliteal arteries); occlusion/restenosis at 12 months for patients with critical ischemia
		**No difference found among interventions** Occlusion/restenosis at 3 weeks and 3 and 6 months (arteries of the pelvis); pseudoaneurysm hematoma and amputation
	LMWH (dalteparin) 2500 IU/d plus ASA 100 mg/d versus ASA 100 mg/d	**Benefit with LMWH plus ASA** Occlusion/restenosis at 12 months for critical ischemia
		**No difference found among interventions** Occlusion/restenosis at 12 months for intermittent claudication

ASA = acetylsalicylic acid, VKA = vitamin K antagonists, DIP = dipyridamole, LMWH = low molecular weight heparin, UFH = unfractionated heparin, AMI = acute myocardial infarction, PGE1 = prostaglandin E1.

For each of the studies included, the objectives and the outcomes assessed that were relevant to this review are demonstrated. At the end, we presented the authors’ opinion in relation to the result.

## ANTIPLATELET AGENTS FOR PREVENTING THROMBOSIS AFTER PERIPHERAL ARTERIAL BYPASS SURGERY

The purpose of the review^8^ was to evaluate the effects of antiplatelet agents for preventing thrombosis in patients who underwent femoropopliteal or femorodistal bypass surgery. The outcomes included graft patency and treatment complications. Sixteen randomized controlled trials (RCTs) (total of 5,683 participants) were included. The main findings were as follows.

### Acetylsalicylic acid (ASA) or ASA plus dipyridamole (DIP) versus placebo or nothing, for venous bypass or prosthetic bypass

**Primary venous graft patency:** benefit through treatment with ASA or ASA plus DIP for outcomes at 12 months (odds ratio, OR: 0.69; 95% confidence interval, CI: 0.48 to 0.99; 2 RCTs; 642 patients); no difference among intervention groups at 1 or 3 months (OR: 0.85; 95% CI: 0.54 to 1.35; 2 RCTs; 342 patients) or 6 or 24 months (OR: 1.03; 95% CI: 0.32 to 3.28; 2 RCTs; 620 patients).

**Primary prosthetic graft patency:** benefit through treatment with ASA or ASA plus DIP for outcomes at 1 month (OR: 0.14; 95% CI: 0.04 to 0.51; 3 RCTs; 157 patients), 3 or 6 months (OR: 0.21; 95% CI: 0.11 to 0.41; 4 RCTs; 122 patients) or 9 or 12 months (OR: 0.19; 95% CI: 0.1 to 0.36; 4 RCTs; 122 patients).

**No differences were found among the groups for the following outcomes:** mortality (evaluated in 4 RCTs with 799 patients); limb amputation (evaluated in 1 RCT with 148 patients); gastrointestinal symptoms (evaluated in 6 RCTs with 952 patients); severe bleeding (evaluated in 2 RCTs with 598 patients); mild bleedings (evaluated in 1 RCT with 148 patients); or wound or graft infection (evaluated in 1 RCT with 549 patients).

### ASA or ASA plus DIP versus pentoxifylline, for venous bypass or prosthetic bypass

**Gastric intolerance:** benefit through use of pentoxifylline (OR: 18.04; 95% CI: 5.07 to 64.17; 1 RCT; 118 patients).

**No differences were found among the groups for the following outcomes:** primary graft patency at 1, 3, 6 or 12 months, mortality, limb amputation, gastric bleeding and dizziness (evaluated in 1 RCT with 118 patients).

### ASA plus DIP versus indobufen, for venous bypass or prosthetic bypass

**No differences were found among the groups for the following outcomes:** primary graft patency at 1, 3, 6, 9 or 12 months (evaluated in 1 RCT with 112 patients).

### ASA or ASA plus DIP versus vitamin K antagonists (VKA), for venous bypass or prosthetic bypass

**No differences were found among the groups for the following outcomes:** primary graft patency at 3, 6, 12 or 24 months and limb amputation (evaluated in 2 RCTs with 2,781 patients); or cardiovascular events and mortality (evaluated in 1 RCT with 2,690 patients).

### ASA plus DIP versus low molecular weight heparin (LMWH), for venous bypass or prosthetic bypass

**Mortality:** benefit through treatment with ASA plus DIP (OR: 0.18; 95% CI: 0.04 to 0.86; 1 RCT; 200 patients).

**No differences were found among the groups for the following outcomes:** primary graft patency at 6 or 12 months (evaluated in 1 RCT with 200 patients).

### Ticlopidine versus placebo, for venous bypass

**Primary venous graft patency:** benefit through use of ticlopidine at 6 months (OR: 0.26; 95% CI: 0.11 to 0.63; 1 RCT; 243 patients) or 12 or 24 months (OR: 0.37; 95% CI: 0.21 to 0.67; 1 RCT; 243 patients); no difference among the intervention groups at 1 month (OR: 3.0; 95% CI: 0.12 to 74.37; 1 RCT; 243 patients).

### ASA versus prostaglandin (E1), for venous bypass

**Early occlusion:** no difference among the intervention groups (evaluated in 1 RCT with 100 patients).

### ASA versus naſtidrofuryl

**No differences were found among the groups for the following outcomes:** primary graft patency, dizziness, gastric bleeding and mortality (evaluated in 1 RCT with 99 patients).

### Clopidogrel plus ASA versus ASA alone

**Primary prosthetic bypass patency:** benefit through use of clopidogrel plus ASA for outcomes at 24 months (OR: 0.53; 95% CI: 0.32 to 0.88; 1 RCT; 253 patients).

**Amputation in prosthetic bypass:** benefit through use of clopidogrel plus ASA (OR: 0.44; 95% CI: 0.21 to 0.91; 1 RCT; 253 patients).

**Minor bleeding in venous bypass:** benefit through use of ASA (OR: 2.46; 95% CI: 1.33 to 4.53; 1 RCT; 598 patients).

**Mild bleeding in venous bypass:** benefit through use of ASA (OR: 5.75; 95% CI: 1.26 to 26.17; 1 RCT; 598 patients).

**No differences were found among the groups for the following outcomes:** amputation for venous bypass and primary venous bypass patency at 24 months (evaluated in 1 RCT with 598 patients); mortality for venous or prosthetic bypasses (evaluated in 1 RCT with 851 patients); minor bleeding for prosthetic bypass, mild bleeding for prosthetic bypass and major bleeding for prosthetic bypass (evaluated in 1 RCT with 253 patients); or major bleeding for venous bypass (evaluated in 1 RCT with 598 patients).

### Conclusions from this review

Treatment with ASA or ASA plus DIP had a positive effect on the patency of patients undergoing venous and prosthetic bypass surgery, especially on prosthetic bypasses, with benefit for outcomes at 1, 3, 6, 9 and 12 months. For venous bypass, the benefit was observed only for the 12-month endpoint. However, this superiority needs further investigation.

A single study evaluated the effect of ticlopidine versus placebo in venous bypass surgery. The results showed improvements at 6, 12 and 24 months after treatment. Thus, the only antiplatelet agent with better effect on venous bypass patency was ticlopidine.

The association of clopidogrel plus ASA versus ASA alone, which was evaluated in a single study, did not show any favorable effects for treatment with ASA plus Clopidogrel for outcomes at 24 months in venous bypass surgery, but favorable results were reported for prosthetic bypass surgery.

The results from this review need to be carefully interpreted since many of the analyses were obtained from a single study and with different dosages.

## ANTITHROMBOTIC AGENTS FOR PREVENTING THROMBOSIS AFTER INFRAINGUINAL ARTERIAL BYPASS SURGERY

This review^9^ aimed to determine the efficacy of antithrombotic agents in patients with peripherical arterial disease (intermittent claudication and critical ischemia) who underwent femoropopliteal or femorodistal bypass surgery. Fourteen RCTs (total of 4,970 participants) were included. The main findings were as follows.

### VKA versus placebo

**Occlusions in venous bypass surgery:** lower number of occlusions (benefit) through treatment with VKA for outcomes at 6 months (OR: 0.4; 95% CI: 0.17 to 0.96; 3 RCTs; 143 patients); no differences among the groups for outcomes at 3 or 12 months (OR: 0.75; 95% CI: 0.49 to 1.14; 4 RCTs; 650 patients) or 24 months or 5 years (OR: 1.0; 95% CI: 0.71 to 1.4; 3 RCTs; 568 patients).

**Occlusions in prosthetic bypass surgery:** lower number of occlusions (benefit) through treatment with VKA for outcomes at 5 years (OR: 0.43; 95% CI: 0.26 to 0.73; 2 RCTs; 140 patients); no differences among the groups for outcomes at 3 or 6 months (OR: 0.87; 95% CI: 0.2 to 2.82; 1 RCT; 33 patients) or 12 or 24 months.

**Limb loss in venous bypass surgery:** benefit through treatment with VKA for outcomes at 5 years (OR: 0.29; 95% CI: 0.14 to 0.6; 2 RCTs; 179 patients); no differences among the intervention groups for outcomes at 3, 6, 12 or 24 months.

**No differences were found among the groups for the following outcomes:** limb loss in prosthetic bypass surgery at 3, 6 or 12 months (evaluated in 1 RCT with 33 patients) or 24 months or 5 years; or mortality in venous or prosthetic bypass surgery at 3, 6 or 12 months (evaluated in 2 RCTs with 268 patients) or 24 months or 5 years.

### VKA versus ASA or ASA plus DIP

**Occlusions in venous bypass surgery:** lower number of occlusions (benefit) through treatment with VKA for outcomes at 3 months (OR: 0.65; 95% CI; 0.46 to 0.92; 2 RCTs; 1,637 patients), 6 or 12 months (OR: 0.65; 95% CI: 0.49 to 0.85; 2 RCTs; 1,640 patients) or 24 months (OR: 0.59; 95% CI: 0.46 to 0.76; 2 RCTs; 1,640 patients).

**Occlusions in prosthetic bypass surgery:** lower number of occlusions (benefit) through treatment with ASA plus DIP for outcomes at 6 months (OR: 1.46; 95% CI: 1.08 to 1.98; 1 RCT; 1,104 patients) or 12 or 24 months (OR: 1.41; 95% CI: 1.11 to 1.8; 1 RCT; 1,104 patients); no differences among the intervention groups at 3 months.

### LMWH versus unfractionated heparin (UFH)

**Occlusions in venous or prosthetic bypass surgery:** lower number of occlusions (benefit) through treatment with LMWH in outcomes at 10 or 30 days (OR: 0.54; 95% CI: 0.33 to 0.9; 2 RCTs; 507 patients); no differences among the intervention groups at 24 hours.

### LMWH versus ASA plus DIP

**Occlusions in venous or prosthetic bypass surgery:** lower number of occlusions (benefit) through treatment with LMWH in outcomes at 6 or 12 months (OR: 0.52; 95% CI: 0.29 to 0.96; 1 RCT; 300 patients).

### LMWH versus placebo

**Occlusions in venous or prosthetic bypass surgery:** no differences among the intervention groups in outcomes at 1, 3 or 12 months (evaluated in 1 RCT with 207 patients).

### UFH versus antithrombin

**Occlusions in venous or prosthetic bypass surgery:** lower number of occlusions (benefit) through UFH in the intraoperative period (OR: 55.0; 95% CI: 1.86 to 1622.6; 1 RCT; 13 patients); no differences among the intervention groups at one month.

### LMWH versus dextran plus heparin

**Early graft occlusion:** no differences among the intervention groups (evaluated in 1 RCT with 277 patients) for venous or prosthetic bypasses.

### Conclusion from this review

This review suggested that the effectiveness of antithrombotic medications depends on the type of graft used (venous or prosthetic).

Patients undergoing venous bypass surgery are likely to benefit more from VKA than from platelet inhibitors, while patients undergoing prosthetic bypass surgery are likely to benefit more from antiplatelets (ASA) than from VKA. To support this information and rule out divergences, studies that are more homogenous, with larger samples and detailed descriptions of the participants’ characteristics, are needed.

Regarding treatment with LMWH versus UFH, the results were marginal and further studies with more patients are needed, for better comparisons.

For LMWH versus ASA plus DIP, better patency was found in the LMWH group, but this advantage was observed for patients with critical ischemia and not for patients with claudication. Further studies are needed in order to confirm these results.

## ANTIPLATELET AND ANTICOAGULANT DRUGS FOR PREVENTION OF RESTENOSIS/REOCCLUSION AFTER PERIPHERAL ENDOVASCULAR TREATMENT

This review^10^ evaluated whether any antithrombotic drug was more effective in preventing restenosis or reocclusion after endovascular treatment, in comparison with any antithrombotic drug, no treatment, placebo or vasoactive drugs. Twenty-two RCTs (3,529 participants in total) were included. The main findings were as follows.

### ASA plus DIP versus placebo

**Occlusions/restenosis:** lower numbers of occlusions/restenosis (benefit) through treatment with ASA 330 mg at 6 months (OR: 0.4; 95% CI: 0.19 to 0.87; 1 RCT; 133 patients). No differences were found with regard to immediate occlusion or primary occlusion at 1, 3 or 6 months (including with different doses of 330 mg) or 12 months.

**Occlusions/restenosis with high or low doses of ASA plus DIP:** no differences among the intervention groups at 1 month (OR 1.45; 95% CI: 0.63 to 3.35; 3 RCTs; 748 patients) or 3, 6, 12 or 24 months.

**Gastrointestinal side effects:** higher number of side effects through treatment with high doses of ASA plus DIP at 12 or 24 months (OR: 1.85; 95% CI: 1.15 to 2.98; 2 RCTs; 575 patients).

**No differences were found among the groups for the following outcomes:** amputations, mortality and bleeding at a puncture site at 1 month (evaluated in 1 RCT with 223 patients).

### ASA plus DIP versus VKA

**Occlusions/restenosis:** no differences among the intervention groups at 1, 3, 6, 12, 24 or 36 months (evaluated in 2 RCTs with 289 patients).

### Clopidogrel plus ASA versus LMWH followed by warfarin

**Occlusions/restenosis:** no differences among the intervention groups at 24 hours or 1, 6, 12 or 18 months (evaluated in 1 RCT with 103 patients).

**Risks of major bleeding:** higher for treatment with LMWH followed by warfarin (OR: 0.08; 95% CI: 0.01 to 0.63; 1 RCT; 103 patients).

### Ticlopidine versus VKA

**Occlusions/restenosis:** no differences among the intervention groups at 12 months (evaluated in 1 RCT with 197 patients).

**Side effects:** higher number of side effects through use of ticlopidine (OR: 6.48; 95% CI: 2.27 to 20.55; 1 RCT; 103 patients). Treatment was suspended for 34% of the patients due to gastrointestinal side effects.

### Cilostazol plus ASA versus ticlopidine plus ASA

**Occlusions/restenosis:** lower numbers of occlusions/restenosis (benefit) through treatment with cilostazol plus ASA for outcomes at 36 months (OR: 0.4; 95% CI: 0.19 to 0.83; 1 RCT; 127 patients); no differences among groups with outcomes at 12 or 24 months.

**No differences were found among the groups for the following outcomes:** amputations, mortality and side effects, all with a follow-up of 36 months (evaluated in 1 RCT with 127 patients).

### LMWH (nadroparin) plus ASA versus UFH plus ASA

**Occlusions/restenosis:** lower numbers of occlusions/restenosis (benefit) through treatment with LMWH (nadroparin) at 3 weeks (OR: 0.3; 95% CI: 0.13 to 0.68; 1 RCT; 110 patients), 3 months (OR: 0.32; 95% CI: 0.15 to 0.7; 1 RCT; 100 patients) or 6 months (OR: 0.16; 95% CI: 0.07 to 0.4; 1 RCT; 110 patients) for femoropopliteal arteries; or 12 months for patients with critical ischemia (OR: 0.15; 95% CI: 0.06 to 0.42; 1 RCT; 79 patients). No differences in occlusions/restenosis were found at 3 weeks or 3 or 6 months for pelvic arteries.

**No differences were found among the groups for the following outcomes:** hematoma < 10 cm, pseudoaneurysms at 3 weeks or amputations at 6 months (evaluated in 1 RCT with 172 patients).

### LMWH (dalteparin) plus ASA versus ASA alone

**Occlusions/restenosis:** lower numbers of occlusions/restenosis (benefit) through treatment with LMWH plus ASA at 12 months for patients with critical ischemia (OR: 0.15; 95% CI: 0.06 to 0.42; 1 RCT; 79 patients). This benefit was not observed for patients with intermittent claudication (OR: 1.73; 95% CI: 0.97 to 3.08; 1 RCT; 196 patients).

### Conclusions from this review

The authors concluded that there was limited evidence to suggest that occlusions/restenosis after six months of endovascular treatment were reduced through use of antiplatelet drugs, in comparison with placebo/controls.

The best evidence points to use of dipyridamole plus ASA, cilostazol plus ASA and low molecular weight heparin (with or without ASA).

Cilostazol plus ASA was superior to ticlopidine, and LMWH plus ASA was superior to ASA, in assessing restenosis/occlusion at 12 months after the start of intervention.

The clinical trials included in this systematic review were small, and the side effects could not be consistently evaluated.

A single study^11^ evaluated the use of ASA plus clopidogrel compared with LMWH plus warfarin, but without evidence of superiority for either of the treatments. Use of ASA plus clopidogrel seems to have off-label indications mainly in the United States. RCTs evaluating use of ASA plus clopidogrel for acute coronary syndrome showed significant reductions in cardiovascular deaths (AMI or stroke), but they showed that this treatment led to increased risk of major bleeding.

## DISCUSSION

This study included three Cochrane systematic reviews.^8^^–^^10^ No evaluation using the GRADE (Grading of Recommendations, Assessment, Development and Evaluation) criteria was done in any of the reviews included in this study. This evaluation is now mandatory for publication of systematic reviews in the Cochrane Library.

The results in **Table 2** can be used for guidance for healthcare professionals and managers, but it needs to be borne in mind that future studies may substantially change the results that have been published so far.

For patients revascularized with prostheses, there was a benefit from use of ASA plus clopidogrel, and especially from use of ASA alone or ASA plus DIP. This benefit was not observed in relation to venous grafts.^8^

For venous bypass surgery, the best results were observed through use of ticlopidine. For both venous and prosthetic bypass surgery, the results need to be carefully interpreted because they were based on few studies.^8^

Patients undergoing venous bypass surgery are likely to benefit more from VKA than from platelet inhibitors. Comparing use of LMWH versus use of ASA plus DIP, better results were found in the LMWH group, but these results were observed in patients with critical ischemia and not in patients with claudication. Further studies are needed to confirm these results.

Patients undergoing prosthetic bypass surgery are likely to benefit more from antiplatelet (ASA) than from VKA.^9^

For patients undergoing endovascular procedures, the best results came from use of ASA plus DIP, from cilostazol plus ASA and from LMWH. Cilostazol plus ASA was superior to ticlopidine; and LMWH plus ASA was superior to ASA.

## CONCLUSION

This study included three Cochrane systematic reviews that provided evidence regarding use of antiplatelets and anticoagulants in lower-limb revascularization in patients with lower-limb PAD. Patients who underwent venous or prosthetic bypass surgery were included, as well as patients who received endovascular treatments.

We noticed that patients undergoing prosthetic graft revascularization presented better outcomes through use of antiplatelet agents. Patients undergoing venous graft revascularization showed better outcomes through use of anticoagulants. In patients undergoing endovascular treatment, use of both antiplatelet and anticoagulant medication proved to be beneficial.

However, the lack of use of the GRADE approach in the reviews included compromised the certainty of our evaluation of the evidence. Updates to these reviews are needed in order to assess the implications of these treatments for clinical practice, among patients with PAD.
